# REGIONAL AND MULTIFACTORIAL PATTERNS OF INFLAMMATORY BOWEL DISEASE IN BRAZIL: A SYSTEMATIC ANALYSIS OF HOT SPOTS IN WESTERN SÃO PAULO AND PARANÁ

**DOI:** 10.1590/S0004-2803.24612026-007

**Published:** 2026-09-21

**Authors:** Abel Botelho QUARESMA, Fabio V. TEIXEIRA, Douglas A. VALVERDE, Adriano A. F. HINO, Aderson OMC DAMIÃO, Paulo Gustavo KOTZE

**Affiliations:** 1 Universidade do Oeste de Santa Catarina, UNOESC, Ambulatório de Coloproctologia e Doenças Inflamatórias Intestinais, Joaçaba, SC, Brasil.; 2 Pontifícia Universidade Católica do Paraná, Pós-graduação em Ciência da Saúde, Curitiba, PR, Brasil.; 3 Clínica GastroSaúde, Marília, SP, Brasil.; 4 Techtrials Healthcare, Data Science, Vinhedo, SP, Brasil.; 5 Universidade de São Paulo, Gastroenterologia, São Paulo, SP, Brasil.; 6 Pontifícia Universidade Católica do Paraná, Ambulatório de Doenças Inflamatórias Intestinais, Unidade de Cirurgia Colorretal, Curitiba, PR, Brasil.

**Keywords:** Inflammatory Bowel Disease, epidemiology, hot spots, urbanization, Brazil, agro-industrialization, Doença Inflamatória Intestinal, epidemiologia, hot spots, urbanização, Brasil, agro-industrialização

## Abstract

**Background:**

Inflammatory Bowel Disease (IBD) is a complex condition influenced by interactions among genetic, environmental, immunological, and behavioral factors. The global rise in IBD, particularly in rapidly urbanizing countries like Brazil, reflects environmental transitions’ impact on disease patterns. Regional disparities in diagnostic coverage and environmental factors highlight IBD “hot spots”, particularly in Western São Paulo and Northwestern Paraná, which share similar socio-economic and environmental characteristics. This study systematically reviews these epidemiological patterns to address gaps and support public health policies.

**Methods:**

A systematic literature review adhering to PRISMA guidelines was conducted to assess Brazilian IBD epidemiology from 2006 to 2026. Data sources included PubMed, SciELO, and LILACS, with Boolean search strategies incorporating controlled vocabulary and free-text terms. Eligible studies provided primary epidemiological data on IBD incidence, prevalence, and associated risk factors. Hot spots were identified through geospatial analysis using Moran’s I index, supported by integrative conceptual models like Directed Acyclic Graphs (DAGs).

**Results:**

The review identified prominent IBD hot spots in Western São Paulo and Northwestern Paraná, characterized by shared urbanization (85%+), agro-industrial activity, and dietary transitions. These regions exhibit increased prevalence due to multifactorial mechanisms, such as genetic predisposition, Westernized diets, reduced environmental microbial exposure, and pesticide-related dysbiosis. However, diagnostic infrastructure disparities highlight underreporting in less developed regions, complicating nationwide comparisons.

**Conclusion:**

Western São Paulo and Northwestern Paraná form a continuous IBD hot spot influenced by urbanization, agro-industrial practices, and environmental exposures. Addressing these challenges requires integrated public health policies focusing on environmental regulation, nutritional education, and improved diagnostic access. Future longitudinal studies should assess gene-environment interactions and further explore underrepresented regions to enhance IBD management and prevention strategies in Brazil.

## HIGHLIGHTS

• The article analized the epidemiological patterns of Inflammatory Bowel Disease (IBD) in Brazil, highlighting “hot spots” in Western São Paulo and Northwestern Paraná, linked to high urbanization, westernized diets, and agro-industrial activities.

• Utilizing systematic review and spatial analysis, the study confirmed the geographic continuity of these areas as major zones of high disease prevalence.

• Multifactorial mechanisms, including environmental exposure, genetic predisposition, and unequal diagnostic infrastructure, were identified as significantly impacting IBD records and management.

• The study emphasizes the need for integrated public policies to address environmental factors, enhance nutritional education, and improve diagnostic access, particularly in underprivileged regions.

## RESUMO

**Contexto:**

A Doença Inflamatória Intestinal (DII) é uma afecção complexa, modulada pela interação entre fatores genéticos, ambientais, imunológicos e comportamentais. A elevação global da incidência de DII, particularmente em países com urbanização acelerada como o Brasil, reflete o impacto da transição ambiental no comportamento epidemiológico da doença. Disparidades regionais na cobertura diagnóstica e em fatores ambientais evidenciam hot spots (áreas de alta prevalência) de DII, notadamente no Oeste Paulista e no Noroeste do Paraná, que compartilham características socioeconômicas e ambientais sobreponíveis. Este estudo revisa sistematicamente tais padrões epidemiológicos para mitigar lacunas de dados e subsidiar políticas de saúde pública.

**Métodos:**

Conduziu-se uma revisão sistemática da literatura, em estrita conformidade com as diretrizes PRISMA, para avaliar a epidemiologia da DII no Brasil entre 2006 e 2026. As bases de dados consultadas incluíram PubMed, SciELO e LILACS, utilizando estratégias de busca booleana com vocabulário controlado (MeSH/DeCS) e termos livres. Os estudos elegíveis forneceram dados epidemiológicos primários sobre incidência, prevalência e fatores de risco associados à DII. Os *hot spots* foram identificados por meio de análise geoespacial empregando o Índice de Moran, corroborados por modelos conceituais integrativos, como os Grafos Acíclicos Direcionados (DAGs).

**Resultados:**

A revisão identificou *hot spots* proeminentes de DII no Oeste Paulista e Noroeste do Paraná, caracterizados por alta taxa de urbanização (acima de 85%), intensa atividade agroindustrial e transição dietética. Essas macrorregiões exibem prevalência aumentada decorrente de mecanismos multifatoriais, incluindo predisposição genética, ocidentalização da dieta, redução da exposição a microbiomas ambientais e disbiose associada à exposição a defensivos agrícolas (agrotóxicos). Contudo, a heterogeneidade na infraestrutura diagnóstica sugere subnotificação em regiões menos desenvolvidas, introduzindo um viés de aferição que complexifica as comparações em âmbito nacional.

**Conclusão:**

O Oeste Paulista e o Noroeste do Paraná configuram um *hot spot* contínuo de DII, influenciado pela urbanização, práticas agroindustriais e exposições ambientais (expossoma). O enfrentamento desses desafios exige políticas de saúde pública integradas, com foco em regulação ambiental, reeducação nutricional e democratização do acesso a métodos diagnósticos. Estudos longitudinais prospectivos são imperativos para elucidar as interações gene-ambiente e investigar regiões sub-representadas, visando otimizar o manejo e as estratégias de prevenção da DII no Brasil.

## INTRODUCTION

Inflammatory Bowel Disease (IBD) is a complex condition with an etiology that involves dynamic interactions between genetic, immunological, environmental, and behavioral factors^1^ ^-^ ^3^. Over the past decades, the global rise in IBD incidence, particularly in countries that have undergone rapid urbanization and socioeconomic transitions, underscores the significant influence of environmental and behavioural exposures on disease risk modulation^4^ ^-^ ^7^. Previous studies have highlighted the impact of globalization and environmental and structural determinants on the evolving epidemiology of IBD in Brazil^8^ ^,^ ^9^.

More recently, temporal trends showing an increasing prevalence of IBD within the public healthcare system have emphasized marked regional disparities associated with diagnostic coverage and environmental factors. These studies have identified epidemiological hot spots in Brazil, with a substantial rise in prevalence in the Southeast region and, more recently, in the South, particularly in Western São Paulo and Northwestern Paraná^10^ ^-^ ^14^. Furthermore, investigations carried out across regions with varying socioeconomic characteristics reinforce the geographic variability in IBD prevalence and incidence patterns in Brazil^15^ ^-^ ^18^. For instance, research conducted in less developed areas, such as the Northeast region of the country, shows lower incidence rates compared to more urbanized and industrialized regions^15^ ^,^ ^18^.

Within the context of the identified epidemiological hot spots, a high-prevalence area has been highlighted in Western São Paulo^10^ ^,^ ^11^. Recent studies suggest that this pattern is replicated in Paraná^13^ ^,^ ^14^, with both regions sharing similar environmental, demographic, and socioeconomic characteristics, such as intense urbanization (above 80%), a robust agro-industrial base, and interconnected internal migration patterns. It is hypothesized that these areas collectively form part of a continuous IBD hot spot.

This study aims at a broader understanding of the epidemiological patterns of IBD in critical regions of Brazil, focusing on the similarities between Western São Paulo and Northwestern Paraná, areas that form a continuous hot spot for IBD as identified in previous studies^12^ ^,^ ^13^. Our review seeks to address knowledge gaps, propose new hypotheses regarding the multifactorial mechanisms underlying IBD, and support the development of public health policies tailored to these regions.

## METHODS

This study consists of a systematic literature review conducted in accordance with the recommendations of the Preferred Reporting Items for Systematic Reviews and Meta-Analyses (PRISMA)^19^. The methodology includes a systematic review of the literature, combined with critical appraisal and synthesis of evidence regarding the epidemiology of IBD in Brazil. The review encompasses factors such as incidence, prevalence, geographic distribution, temporal trends, and risk factors over the period from 2006 to 2026. This study is grounded in the works of Quaresma et al. (2022) and da Luz Moreira et al. (2022), which identified significant IBD hot spots in Western São Paulo and Northwestern Paraná - regions exhibiting higher disease prevalence compared to neighbouring areas^12^ ^,^ ^13^.

The bibliographic search was conducted in a comprehensive and reproducible manner with the aim of identifying all relevant studies published on the epidemiology of IBD in Brazil. The PubMed/MEDLINE, SciELO, and LILACS databases were consulted. Search strategies were tailored to the specific requirements of each database and included controlled vocabulary terms (e.g., MeSH and DeCS), along with free-text terms to increase both the sensitivity and precision of the search. Descriptors were used in English and combined with Boolean operators (“AND” and “OR”). Examples of search strings include: “Inflammatory bowel disease” AND “epidemiology” AND “Brazil”; “IBD” AND (“incidence” OR “prevalence”) AND “Brazil”; “Crohn disease” AND “epidemiology” AND “Brazil”; and “Ulcerative colitis” AND “risk factors” AND “Brazil”.

The inclusion criteria specified that eligible studies would be Brazilian observational epidemiological research reporting primary epidemiological data in IBD. Publications were required to be conducted between 2006 and 2026, written in English, and to have clearly described methodologies. These studies had to present data on IBD incidence, prevalence, geographic distribution, or associated risk factors. Exclusion criteria eliminated case reports, case series without significant population-level relevance, experimental studies, and clinical trials focused solely on treatment methodologies. Furthermore, studies with inadequate methods or incomplete data were excluded, as were duplicate articles or preliminary versions of studies subsequently published in full.

The study selection process followed two independent stages. The first stage involved screening titles and abstracts, conducted by two independent reviewers (ABQ and FVT) to discard irrelevant articles. The second stage entailed a thorough review of the full-text articles to rigorously apply the inclusion and exclusion criteria. In cases of disagreement between the reviewers, conflicts were resolved through consensus or intervention by a third reviewer (PGK). The entire process was documented in a PRISMA flow diagram. Data extracted from the included studies were systematically organized and encompassed information such as author(s), journal and year, region and state of Brazil, study population and sample size, and associated risk factors.

### Concept of hot spot

The concept of prevalence hot spots refers to the identification of geographic areas with a high concentration of prevalent cases of a health condition - in this case, IBD - based on spatial statistical analysis of geographic patterns and interactions between localities. The analysis utilizes data from the Brazilian Public Health System (DATASUS) as well as tools such as ArcGIS, which applies Moran’s I spatial autocorrelation index to identify prevalence clusters.

Hot spots represent municipalities or regions where the prevalence of IBD is significantly higher. Statistical significance is evaluated using z-scores and p-values with 95% confidence intervals, and a Moran’s I value close to +1 confirms high spatial dependence^13^. The primary objective of identifying hot spots is to guide strategies for disease management, prevention, and treatment in areas with a high disease burden, thus optimizing resource allocation in regions with the greatest impact. This approach enables public health actions and resources to be prioritized in locations most affected by the burden of IBD.

### Complementary conceptual approach

Integrative conceptual models, such as Directed Acyclic Graphs (DAGs), were employed to assist in interpreting the multifactorial relationships between genetics, environment, urbanization, access to healthcare services, and the observed prevalence of IBD in Brazil.

## RESULTS

**FIGURE 1:**
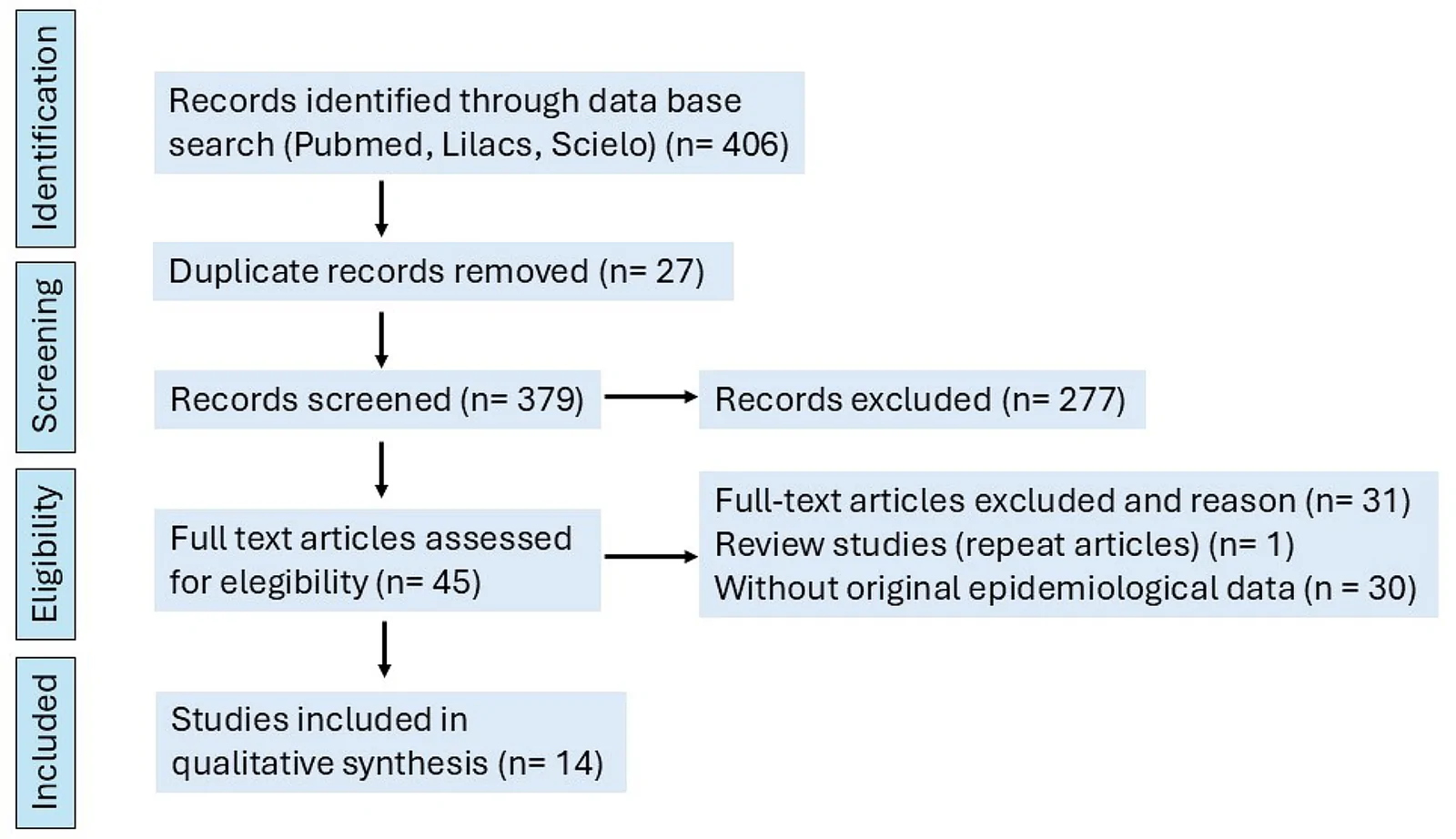
PRISMA Flowchart.

The process of study selection and inclusion of articles was conducted following the PRISMA framework and is summarized in Figure 1.

### Studies evaluated

Table 1 shows the main characteristics of the included studies. Prevalence rates per 100,000 inhabitants according to each study: de Brito 2023 (NR), Cassol 2022 (DII: 9.51; DC: 6.89; RCU: 2.62), Dall`Oglio 2020 (NR), Fróes 2024 (NR), Gasparini 2018 (DII: 52.6; DC: 24.3; RCU: 28.3), Lima Martins 2018 (DII: 38.2; DC: 14.1; RCU: 24.1), MARTINS 2021 (DII: 30.29; DC: 15.23; RCU: 15.06), da Luz Moreira 2022 (DII: 80.0; DC: 36.0; RCU: 46.0), Parente 2015 (DII: 12.8; DC: NR; RCU: NR), QUARESMA 2022 (DII: 100.1; DC: 33.7; RCU: 56.5), RENUZZA 2022 (DII: 58.88; DC: 15.84; RCU: 42.99), Salgado 2020 (NR), VICTORIA 2009 (DII: 22.61; DC: 5.65; RCU: 14.81) and Zaltman 2021.

**TABLE 1:** Main characteristics of the included studies.

Author	Journal	Possible hot spots	Potential risk factors	Cases	Period
**Victoria** **2009** ^10^	Arq Gastroenterol	The study focused exclusively on the central-western region of SP, noting it is less industrialized.	1. Concentration of cases in urban areas.	115 cases	1986 to 2005
			2. Lifestyle changes.		
			3. High proportion of white individuals: 91.07%.		
**Parente** **2015** ^15^	World J Gastroenterol	Teresina was identified as a possible functional hot spot.	1. Increased urbanization and lifestyle changes.	252 cases	2011 to 2012
			2. Higher educational attainment and concentration in urban areas.		
			3. 11.5% of patients had a family history of the disease.		
			4. 86% lived in urban areas with better healthcare access, while 14% were in rural areas.		
**Gasparini** **2018** ^11^	Clin Exp Gastroenterol.	No “areas of concentration” were identified compared to other parts of the state.	1. Improved diagnosis.	22,638 cases	2012 to 2015
			2. Improved access to the SUS (Unified Health System)		
			3. The most urbanized population in the country: greater exposure to risk factors.		
**Lima Martins** **2018** ^16^	BMC Gastroenterol	The study treated the area as a single unit, without highlighting high-prevalence regions.	1. More urbanized regions due to lifestyle changes.	1,048 cases	2012 to 2014
			2. Reduced exposure to environmental microbes.		
			3. Increased contact with other factors (ultra-processed foods and pollution).		
			4. Westernized diets.		
			5. European colonization, genetically more vulnerable.		
**Salgado** **2020** ^20^	Arq Gastroenterol	It identifies the Southeast (SP, MG, and RJ) as the main hotspot region.	1. Urban living and poor housing conditions linked to UC.	1,456 cases	2015 to 2017
			2. Lack of childhood infectious diseases.	CD: 548,	
			3. Treated water: higher CD risk; untreated water: higher UC risk.	UC: 492,	
			4. Family history as the main risk factor for both conditions.	healthy controls: 416	
			5. Smoking: Ex-smokers are at higher risk only for UC.		
			6. Low family income (up to 3 minimum wages).		
			7. Higher educational level: associated with CD.		
			8. Appendectomy: increased risk for CD; protective for UC.		
**Martins** **2021** ^21^	Arq Gastroenterol	The study identified IBD prevalence in Uberlandia (western MG) at the Federal University Hospital but did not compare regions for hotspots.	1. Women predominated in both diseases: 64% CD (*P*=0.011) and 62% UC (*P*=0.026).	183 cases.	1999 to 2014
			2. Majority of patients were white.		
			3. Urban residence: 100% of CD and 97% of UC patients.	92 CD (50,3%)	
			4. Smoking increased the risk for CD, but its protective effect for UC was unconfirmed.	(91 UC 49,7%)	
**Zaltman** **2021** ^22^	World J Gastroenterol	The Southeast showed the highest prevalence of CD, but the study did not compare regions to identify hotspots.	1. Female gender. 2. Higher education levels and employment rates.	407 cases	2016 to 2017
			3. Family history of IBD.	264 CD	
			4. Smoking.	143 UC	
Dall`Oglio 2020^23^	São Paulo Med J.	The evaluation of hotspots was not the aim of the study, which exclusively assessed the city of Caxias do Sul (RS).	1. Female predominance: 71.6% were women.	67 cases	2016
			2. Age: Mean age of 46.5±16.2 years.	UC: 47,	
			3. Smoking.	CD: 16, IBD: 4	
**da Luz Moreira** **2022** ^12^	Inflamm Bowel Dis.	Higher prevalence in the South and Southeast, with hotspots in SP (northwestern areas), SC, ES, and DF.	1. Socioeconomic and demographic factors: HDI, white skin, and urbanization.	162,894 cases	2008 to 2017
			2. Environmental impact: Biodiversity loss, diet westernization, and sun exposure.	UC: 95,618	
			3. Reduction in infections in young people.	CD: 67,276	
**Cassol** **2022** ^17^	World J Gastroenterol	The prevalence was higher in the metropolitan region of Porto Alegre (RS), surpassing other areas of the state.	1. Genetic factors.	1,082 cases	2014 to 2019
			2. Highly urbanized environments.		
			3. Access to reference centres facilitates diagnosis.	784 CD	
			4. Diet westernization and lifestyle changes.	298 UC	
**Renuzza** **2022** ^14^	Arq Gastroenterol	All phenotypes predominated in the eastern region (Curitiba - PR): IBD 39.6% (2,674), CD 43.0% (781), and UC 38.4% (1,893).	Factors associated with urban lifestyle, such as greater industrialization and urbanization, with potential genetic predisposition and other environmental factors. Regions with an agricultural economy show a lower frequency of IBD.	6,748 cases	2010 to 2019
**Quaresma 2022** ^13^	Lancet Reg Health Am.	Areas with higher prevalence: SP, PR, SC, and RS. The Moran’s I spatial autocorrelation index confirmed a south-north gradient.	1. Urbanization and Socioeconomic Development: Increased prevalence in urbanized and developed regions.	212,026 cases.	2012 to 2020
			2. Genetic Factors: Colonization by European immigrants in the South and Southeast states.	UC: 119,700	
			3. Westernization of diet and lifestyle changes.	CD: 71,321	
			4. Improved healthcare access. 5. Sun exposure differences may influence prevalence.	UIBD: 21,005	
**de Brito** **2023** ^18^	Clin Exp Gastroenterol	Recife, Natal, and João Pessoa stand out as hotspots in the Northeast, concentrating 35% of the patients.	1. Increasing urbanization in the Northeast.	571 cases	2020 to 2021
			2. Shift to westernized diets.		
			3. Reduction in microbial diversity.		
			4. Low income and deficiencies in the healthcare system	355 (62%) UC	
			5. Family history of IBD in 12% of patients. 6. Smoking: greater impact on UC	216 (38%) CD	
**Froes** **2024** ^24^	Sci Rep.	Southeast: 72.3% of patients;	1. Urbanization	1179 cases	2020 to 2022
		Central-West: 13.8%;	2. Improved medical infrastructure	46,6% UC,	
		Northeast: 6.9%; South: 4.9%;	3. Greater access to specialized healthcare services	44,2% CD,	
		North: 1.9%.		0,9% UIBD	

Rio Grande do Sul (RS); Santa Catarina (SC); Paraná (PR); São Paulo (SP); Minas Gerais (MG); Rio de Janeiro (RJ); Espírito Santo (ES); Federal District (DF); Inflammatory Bowel Disease (IBD); Ulcerative Colitis (UC); Crohn’s Disease (CD); Unclassified Inflammatory Bowel Disease (UIBD); NR: not reported.

### Synthesis of evidence

The findings of this review highlighted the primary epidemiological hot spots for IBD located in the Western region of São Paulo and Northwestern Paraná. These areas stood out due to their significant size, geographical continuity, and marked prevalence of the disease in the Southern and Southeastern regions of Brazil. While other smaller hot spots were identified in specific areas within these regions, the focus remained on these two extensive and contiguous hot spots because of their notable epidemiological relevance and shared characteristics, which make them critical for future research.

The analysis of factors associated with IBD in Brazil reveals a multifactorial scenario wherein environmental, behavioural, genetic, and structural factors interact to amplify the risk and prevalence of the disease. The inclusion of Northwestern Paraná as an extension of the hot spot initially identified in Western São Paulo suggests a geographic continuity characterized by shared epidemiological, demographic, and structural features.

Improvements in administrative records within Brazil’s SUS (Unified Health System), specifically the SIH/SUS and SIA/SUS databases, over recent decades have resulted in more detailed diagnoses in these regions. However, these advancements may introduce a surveillance bias, where the higher number of reported cases is driven by greater access to available infrastructure rather than a true increase in incidence rates^13^. These regional differences highlight the importance of distinguishing true prevalence from recorded prevalence in future studies.

**FIGURE 2:**
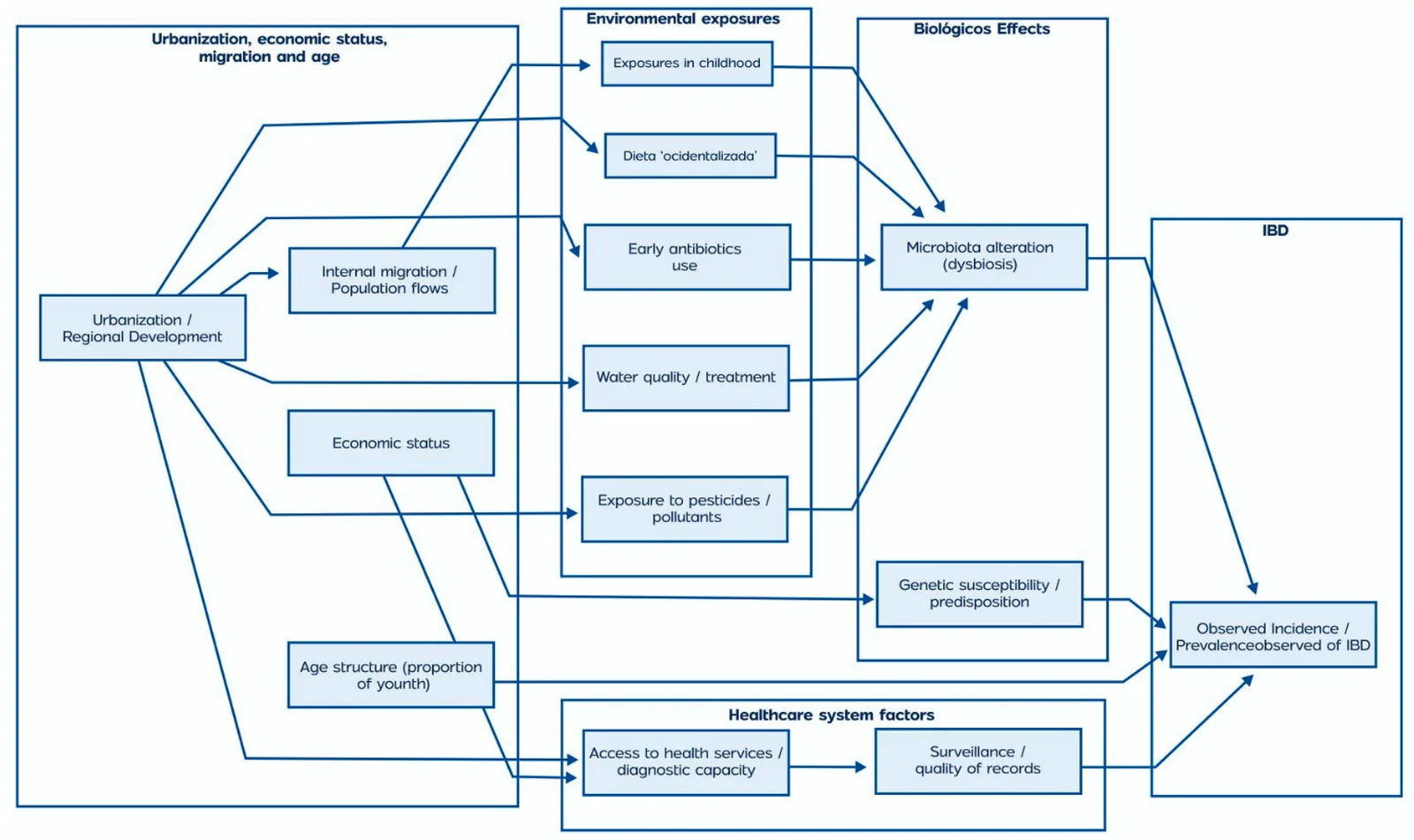
Directed Acyclic Graph (DAG) illustrating how various factors are interconnected and influence a specific outcome within the hot spot located in Western São Paulo and Paraná. Source: Epiwriting Knowledge Base (Techtrials®).

The interaction between genetic and environmental factors, enhanced by structural variables, contributes to the regional concentration of IBD. Specific environmental exposures, such as intensive agro-industrial activity and the adoption of Westernized diets, distinguish these regions from other parts of Brazil. The main factors involved are illustrated in Figure 2.

## DISCUSSION

IBD in Brazil reflects a multifactorial framework in which environmental, biological, structural, and behavioral factors interact in complex ways to shape regional prevalence patterns^13^. Epidemiological hot spots, such as those identified in Western São Paulo and Northwestern Paraná, are critical areas for studying these interactions, as they combine intense agro-industrial activity, rapid urbanization, and dietary changes^25^ ^-^ ^27^.

These characteristics make these regions particularly susceptible to the progression of IBD, while also highlighting structural inequalities and research gaps that need to be addressed to advance disease management. The genetic admixture of the Brazilian population provides a unique epidemiological landscape, in which genetic factors associated with IBD may manifest differently depending on regional environmental conditions^28^ ^-^ ^30^. However, gene-environment interactions remain underexplored in Brazil, limiting the ability to identify specific genomic variants associated with susceptibility to or protection against IBD. This gap is particularly relevant in regions like Northwestern Paraná and Western São Paulo, where environmental exposures, such as intensive pesticide use, interact with local population genetics exacerbating inflammatory processes^7^ ^,^ ^31^.

Recent studies, such as da Luz Moreira et al*.* (2022), emphasize that biodiversity loss and chemical impacts associated with pesticide use can significantly contribute to changes in human gut microbiota in these agro-industrialized regions^12^. Dysbiosis induced by these exposures is a central mechanism in IBD, reflecting imbalances between beneficial and pathogenic gut bacteria, ultimately leading to chronic inflammatory responses in the gastrointestinal tract^25^ ^,^ ^26^. Such environmental exposures underscore the importance of investing in environmental regulatory policies and public awareness strategies to mitigate the population’s exposure to hazardous agents^32^.

Regions like Western São Paulo and Northwestern Paraná share urbanization rates above 85%, a critical factor contributing to the increasing IBD prevalence in these areas (IBGE 2010). Rapid urbanization is directly associated with reduced exposure to diverse environmental microbiota, significantly impacting immune system balance and favoring inflammatory conditions. This aligns with the “hygiene hypothesis”, which speculate that lower exposure to natural microorganisms in urban settings disrupts healthy immune development, increasing susceptibility to inflammatory diseases^32^ ^-^ ^34^.

Urbanization also accelerates dietary shifts, replacing traditional fiber-rich diets with Westernized diets dominated by ultra-processed foods, high in saturated fats and refined sugars. Studies by Hou et al. (2011) and Narula et al. (2021) demonstrated that these dietary changes increase intestinal permeability, creating a favorable environment for chronic inflammation to develop^35^ ^,^ ^36^. Consequently, more urbanized areas, such as Southeastern and Southern Brazil, experience higher IBD prevalence compared to more rural or less industrialized regions. These findings reinforce the significance of nutritional transitions as a key component in the emergence of hot spots in Brazil.

The intensive agro-industrial model prevalent in Western Paraná and Western São Paulo represents a critical environmental factor for IBD epidemiology. High pesticide density, coupled with monoculture practices, reduces ecosystem biodiversity and directly impacts gut health in exposed populations^12^ ^,^ ^13^. Widely used chemicals, such as pesticides and fertilizers, not only affect human microbiota but also contaminate soil, water, and air, increasing the risk of dysbiosis among local populations. These effects can accumulate over time, exacerbating inflammatory processes and contributing to IBD progression^7^ ^,^ ^37^.

International studies conducted in agricultural hot spots, such as those in Israel, China, and Hong Kong, emphasize the importance of monitoring environmental impacts on exposed populations^32^ ^,^ ^38^. However, there is a lack of Brazilian longitudinal studies correlating these factors with diagnostic markers and the clinical progression of IBD. Such studies are essential to fully understand the interaction between agricultural industrialization and public health.

Structural inequalities in the Brazilian healthcare system directly affect IBD mapping, particularly in the North and Northeast regions, which face insufficient infrastructure for adequate diagnosis. In these areas, underreporting of cases is high, primarily due to the lack of gastroenterology specialists and limited access to diagnostic exams such as colonoscopies^13^ ^,^ ^39^. Conversely, the South and Southeast regions stand out with more developed healthcare systems, greater availability of advanced services, and higher concentrations of specialists. These factors increase captured prevalence rates but also introduce the so-called “surveillance bias”^32^.

This bias highlights the importance of interpreting epidemiological data carefully, as higher numbers in privileged regions reflect both real prevalence and favourable conditions for diagnosis^12^. Additionally, priority regions for studies and policy actions remain underexplored, limiting the overall understanding of the extent of IBD in Brazil.

A deeper understanding of the determinants of IBD in Brazil highlights the need for cross-sectoral interventions that integrate health, environment, and public policy^8^ ^,^ ^12^ ^,^ ^13^. First, stricter regulations on the use of pesticides and chemical fertilizers are critical in agro-industrialized regions such as Western Paraná and São Paulo^12^ ^,^ ^14^. Transitioning to more sustainable agricultural practices could significantly reduce the environmental impact on the health of local populations.

In parallel, educational programs aimed at improving nutritional quality are crucial, particularly in urbanized regions. Encouraging the consumption of high-fiber and natural foods while reducing dependency on ultra-processed products can mitigate the adverse effects of dietary transitions on gut health^24^.

Additionally, improving diagnostic access in less developed regions of Brazil is essential, requiring investment in expanding medical infrastructure, training healthcare professionals, and creating integrated epidemiological surveillance systems^14^ ^,^ ^18^. These measures would generate more robust, representative epidemiological data, making them valuable for formulating effective prevention and management policies.

These findings reinforce the hypothesis of a continuous hot spot in Western São Paulo and Paraná, highlighting the urgency of integrated and interdisciplinary studies to uncover causal mechanisms^13^. They also provide a solid foundation for formulating public health policies aimed at mitigating the growing impacts of IBD on the Brazilian population.

This review has some critical limitations that must be considered. The methodological heterogeneity of the analysed studies complicates the comparability between regions and undermines the precise interpretation of both prevalence data and factors associated with IBD. Furthermore, a surveillance bias is evident: regions with better healthcare infrastructure, such as the South and Southeast, tend to report more diagnoses, while under-resourced areas, such as the North and Northeast, suffer from underreporting. Another significant limitation is the lack of national longitudinal studies that connect environmental exposures, such as pesticide use, with long-term clinical outcomes. There is also a research concentration in high-prevalence regions, especially in the Southeast, leaving rural and less industrialized areas underexplored. Data on the gut microbiota in Brazilian populations are also scarce, despite dysbiosis being recognized as a central mechanism in IBD. Additionally, the exploration of genetic factors remains insufficient, even with Brazil’s unique genetic admixture. This limitation restricts the understanding of gene-environment interactions in the national context. Recognizing these limitations is essential for developing more equitable and effective strategies to manage IBD in Brazil. Addressing these research gaps could promote better health and quality of life for affected populations.

In summary, the regional prevalence patterns of IBD in Brazil provide a clear depiction of the possible interaction among environmental, genetic, and structural factors. The continuous hot spot identified in Western São Paulo and Paraná serves as a robust example of how multifactorial determinants shape the distribution of complex diseases. Overcoming these limitations requires greater uniformity in study designs, expanded access to diagnostic services across all regions, and the strengthening of initiatives focused on environmental sustainability and nutritional education.

**FIGURE 3:**
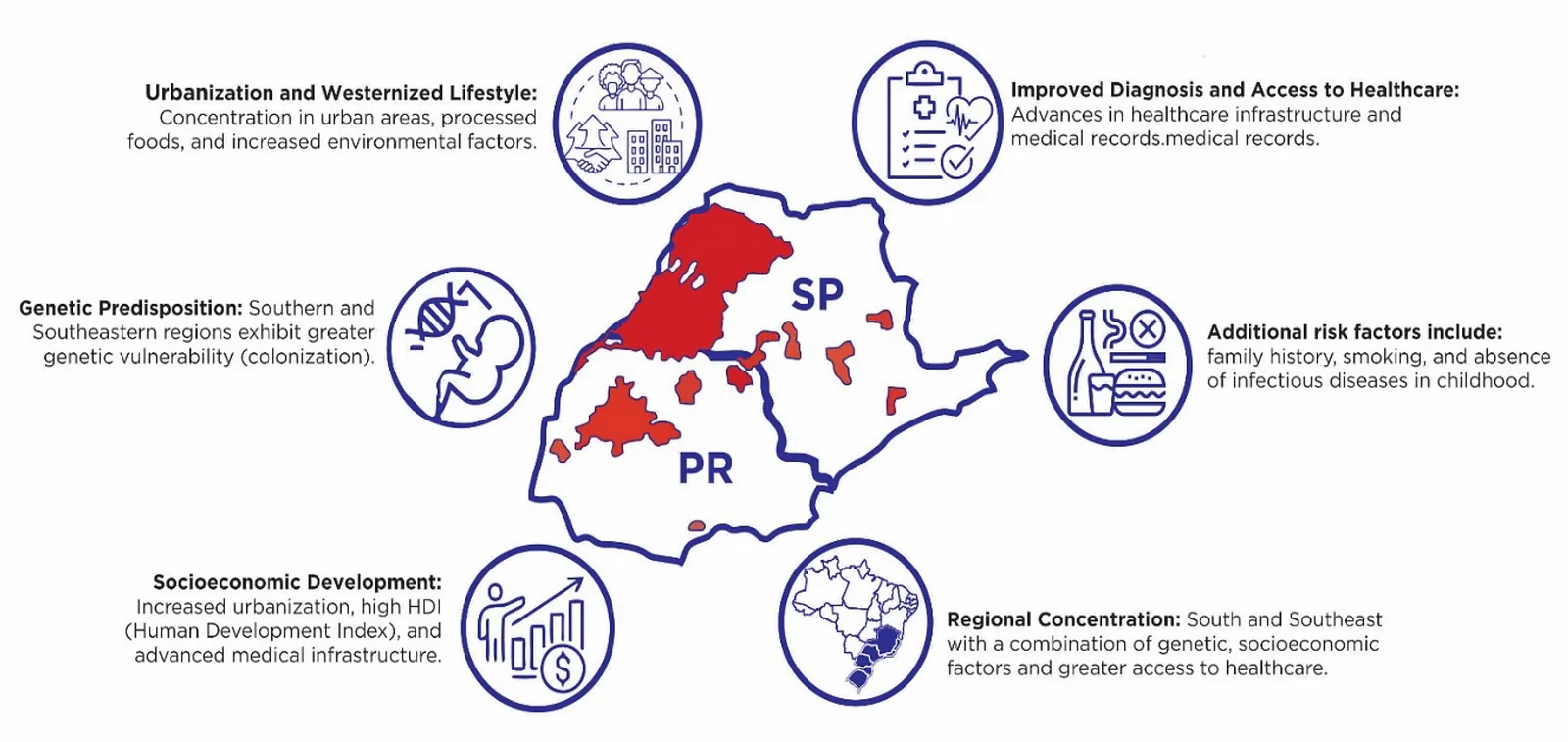
The main risk factors associated with and the distribution of the prevalence hot spot of IBD in the states of Paraná and São Paulo. Adapted from da Luz Moreira et al. 2022 and Quaresma et al. 2022.

The interdisciplinary approach suggested here positions Brazil as a unique setting for innovative studies that integrate genetics, biology, and epidemiology to develop more effective interventions against IBD. Promoting integrated epidemiological surveillance systems and prioritizing cross-sectoral public health initiatives are imperative to addressing the persistent and emerging challenges associated with this condition (Figure 3)^7^.

## Data Availability

Data available upon request.
